# Recent advances in understanding and managing resistant/refractory hypertension

**DOI:** 10.12688/f1000research.21669.1

**Published:** 2020-03-09

**Authors:** Michael Doumas, Konstantinos P Imprialos, Manolis S Kallistratos, Athanasios J Manolis

**Affiliations:** 1 2nd Propedeutic Department of Internal Medicine, Aristotle University of Thessaloniki, Thessaloniki, Makedonia, 54250, Greece; 2 VAMC and George Washington University, Washington, USA; 3 Cardiology, Asklepeion General Hospital, Athens, Attiki, 16673, Greece

**Keywords:** resistant hypertension, spironolactone, doxazosin, bisoprolol, clonidine, renal sympathetic denervation, iliac anastomosis, carotid baroreceptor activation therapy

## Abstract

The management of resistant hypertension presents several challenges in everyday clinical practice. During the past few years, several studies have been performed to identify efficient and safe pharmacological and non-pharmacological options for the management of such patients. The Spironolactone versus placebo, bisoprolol, and doxazosin to determine the optimal treatment for drug-resistant hypertension (PATHWAY-2) trial demonstrated significant benefits with the use of spinorolactone as a fourth-line drug for the treatment of resistant hypertension over doxazosin and bisoprolol. In addition, recent data support that spironolactone may demonstrate superiority over central acting drugs in such patients, as well. Based on the European guidelines, spironolactone is recommended as the fourth-line drug option, followed by amiloride, other diuretics, doxazosin, bisoprolol or clonidine.  Among several device-based approaches, renal sympathetic denervation had fallen into hibernation after the disappointing results of the Renal Denervation in Patients With Uncontrolled Hypertension (SYMPLICITY HTN) 3 trial. However, the technique re-emerged at the epicenter of the clinical and research interest after the favorable results of three sham-controlled studies, which facilitated novel catheters and techniques to perform the denervation. Significant results of iliac anastomosis on blood pressure levels have also been demonstrated. Nevertheless, the technique-related adverse events resulted in withdrawal of this interventional approach. Last, the sympatholytic properties of the carotid baroreceptor activation therapy were associated with significant blood pressure reductions in patients with resistant hypertension, which need to be verified in larger controlled trials. Currently device-based approaches are recommended only in the setting of clinical trials until more safety and efficacy data become available.

## Introduction

The prevalence of resistant hypertension ranges from 5 to 30% on the basis of the definition used by relevant studies ^1^. However, the true prevalence of resistant hypertension after applying a strict definition and having excluded causes of pseudo-resistant hypertension is less than 10% of the patients with treated hypertension ^1^. Importantly, resistant hypertension is related with higher risk for cardiovascular morbidity and mortality, chronic kidney disease, and other hypertension-mediated target organ damage ^2^.

Based on the European guidelines, resistant hypertension is defined as the failure to reduce systolic or diastolic blood pressure (BP) levels (or both) below 140 and 90 mm Hg, respectively, despite treatment with optimal doses (or best-tolerated doses) of an appropriate therapeutic strategy with the triple combination of an angiotensin-converting enzyme inhibitor or an angiotensin receptor blocker with a calcium channel blocker and a thiazide/thiazide-type diuretic. As stated in the guidelines, home or ambulatory BP measurements should be used to confirm inadequate BP control, and exclusion of pseudo-resistant hypertension and secondary hypertension is mandatory to establish the diagnosis ^1^.

In the previous European guidelines (2013), the use of mineralocorticoid receptor antagonists, amiloride, and the alpha-1 blocker doxazosin were considered for the management of resistant hypertension ^3^. During the past few years, several studies in resistant hypertension, and especially the spironolactone versus placebo, bisoprolol, and doxazosin to determine the optimal treatment for drug-resistant hypertension (PATHWAY-2) trial ^4^, resulted in changes in the recommendations for the management of resistant hypertension. Specifically, the addition of “low-dose spironolactone to existing treatment, or the addition of further diuretic therapy if intolerant to spironolactone, with either eplerenone, amiloride, higher-dose thiazide/thiazide-like diuretic or a loop diuretic, or the addition of bisoprolol or doxazosin” is now recommended ^1^. Similarly, the consensus document of the American Heart Association for the management of resistant hypertension recommends the use of either spironolactone or eplerenone as a fourth-line agent, followed by a beta-blocker, a dual beta- and alpha-blocker, clonidine, or diltiazem ^5^. The purposes of this review are to report and critically discuss the findings of recent studies that resulted in a stronger recommendation for the treatment of resistant hypertension and to report treatments under investigation that could prove to be useful in such patients.

## Exclusion of pseudo-resistant hypertension

The exclusion of pseudo-hypertension is of paramount importance for the establishment of an accurate diagnosis. BP is often measured inaccurately; wrong-sized cuffs, measurement of BP only once, placement the cuff over the patient’s clothes, and wrong position of the patient are common mistakes performed in everyday clinical practice ^1^. A 2016 study demonstrated that readings performed in a routine triage setting were higher than the readings performed by trained physicians and resulted in a misdiagnosis of uncontrolled resistant hypertension in 33% of the patients ^6^. Under-treatment is also a common cause of pseudo-resistant hypertension, and studies indicate that a lack of BP control is often attributable to the absence of treatment intensification ^7^.

Another important cause of pseudo-resistant hypertension is poor medication adherence. The recent Renal Sympathetic Denervation as a New Treatment for Therapy Resistant Hypertension (SYMPATHY) trial examined drug adherence with the detection of drug concentrations in blood samples in patients with uncontrolled hypertension on three or more anti-hypertensive drugs or with documented intolerance to two or more of the four major anti-hypertensive drug classes; 16% were non-adherent and 52% were poorly adherent ^8^.

## Exclusion of other causes contributing to resistant hypertension

Lifestyle factors such as excessive alcohol and salt intake contribute to the presence of resistant hypertension. Large amounts of alcohol consumption (three or more drinks per day) have an important dose-related effect on BP levels in both normotensive and hypertensive patients ^9^. Abstinence in heavy alcohol drinkers may decrease 24 hours systolic and diastolic ambulatory BP levels by up to 7.2 and 6.6 mm Hg, respectively ^10^. Usually, patients with resistant hypertension present an average sodium intake that exceeds 10 g per day ^11^. Salt not only increases BP levels but also blunts the anti-hypertensive effect of the BP-lowering drugs ^12^. In salt-sensitive patients (elders, African-Americans, and patients with chronic kidney disease), these effects are much more pronounced ^13^. Moreover, obesity and increased body mass index in general increase significantly BP levels ^1^. The mechanisms that induce hypertension in those patients include the activation of sympathetic nervous system and renin–angiotensin–aldosterone system and also insulin resistance and impaired sodium excretion ^14^. The adoption of current European Society of Cardiology/European Society of Hypertension guidelines on lifestyle changes may significantly decrease BP levels in those patients and contribute to BP control.

**Figure 1.**
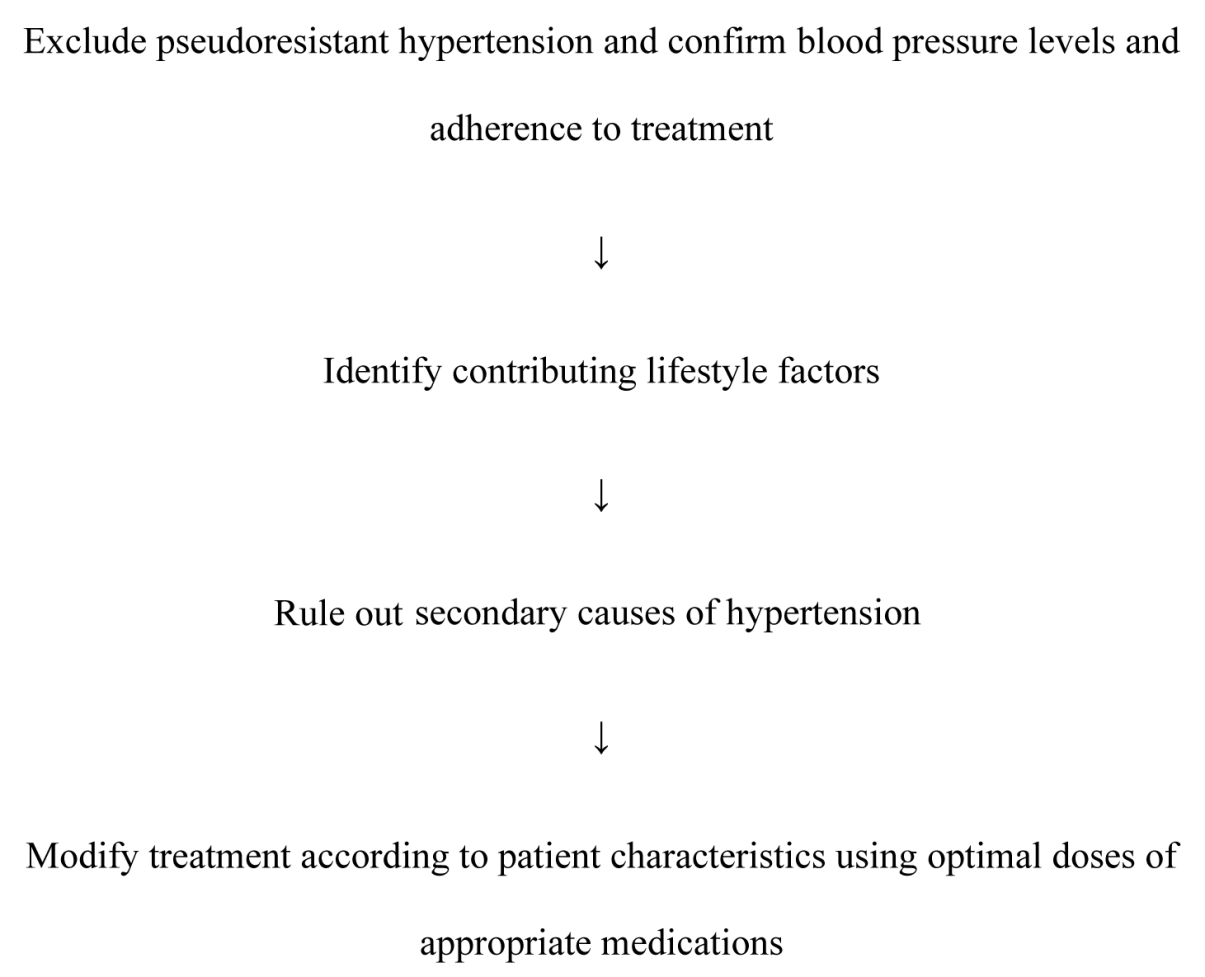
Proposed work-up for patients with resistant hypertension.

Finally, several drugs and substances may increase BP levels. Non-steroidal anti-inflammatory drugs (NSAIDs) represent probably the most common agents in terms of worsening BP control ^1^. The use of NSAIDs not only increases BP levels but also can blunt the effect of various anti-hypertensive drugs such as diuretics, angiotensin-converting enzyme inhibitors, and angiotensin receptor blockers ^1^. The hypertensive effect of NSAIDs is more pronounced in patients with chronic kidney disease ^1^. Other substances that can increase BP levels are decongestants and stimulant agents used for weight loss and also contraceptives, cyclosporine, erythropoietin, and cortisone that increase BP levels mainly through fluid retention. A proposed work-up for patients with resistant hypertension is shown in Figure 1.

## Recent advances in pharmacological therapy

### Data from clinical studies of spironolactone versus adrenergic blockers

The landmark PATHWAY-2 study found significant benefits with the use of spironolactone in patients with resistant hypertension on a standard three-drug therapy with an angiotensin-converting enzyme inhibitor or an angiotensin receptor blocker, amlodipine, and indapamide ^4^. The study was a double-blind four-way crossover study that assessed the use of spironolactone (25 or 50 mg) versus bisoprolol (5 to 10 mg), doxazosin (5 to 10 mg), or placebo. To exclude non-adherence, the study was monitored with pill count and measurement of serum angiotensin-converting enzyme activity. Spironolactone was superior to both active treatments and placebo, and mean reductions in BP were 8.70, 4.48, and 4.03 mm Hg with spironolactone, bisoprolol, and doxazosin, respectively. Importantly, about 60% of the spironolactone users achieved BP control versus 43.3% of the bisoprolol and 41.5% of the doxazosin users ^4^.

Apart from the beneficial effect of spironolactone in patients with resistant hypertension, the PATHWAY-2 study offered three important findings. First, despite the superiority of spironolactone over bisoprolol and doxazosin, the use of the latter two drugs was associated with significant reductions in BP compared with placebo. Thus, the European guidelines recommend the use of bisoprolol and doxazosin for the treatment of resistant hypertension when spironolactone is contraindicated or not tolerated ^1^. Second, uptitration of spironolactone dose from 25 to 50 mg resulted in a greater reduction in BP at week 12 of the study. The BP-lowering effect of spironolactone uptitration was higher compared with the corresponding increases in the dose of either bisoprolol or doxazosin (−3.86 mm Hg with spironolactone versus −0.88 mm Hg for doxazosin, −1.49 mm Hg for bisoprolol, and −0.68 mm Hg for placebo). Last, while spironolactone reduced BP levels irrespective of renin levels, an enhanced benefit in patients with suppressed renin levels was observed, and there was up to a 20 mm Hg reduction in home BP in patients with the lowest renin levels ^4^.

Important clinical information for the management of resistant hypertension arose from three recent substudies of the PATHWAY-2 trial. In the first substudy, the plasma aldosterone, renin, and aldosterone-to-renin ratio were assessed as predictors of home systolic BP response in 126 patients. Plasma aldosterone-to-renin ratio and plasma renin levels were found to be predictors of BP response to spironolactone. In the second one, the impact of each drug on the thoracic fluid content (an index of fluid retention) and vascular resistance was examined (226 patients). Thoracic fluid content was significantly reduced (by 6.8%) from baseline with spironolactone but not with the other active treatments. Given the overall outcomes of the PATHWAY-2 study, this finding supports the theory that patients with resistant hypertension are characterized by volume overload, secondary to aldosterone excess, explaining the greater benefits observed with spironolactone ^1^. In the third substudy, the effect of amiloride on systolic BP was examined in a 6- to 12-week open-label run-out phase, in which patients on spironolactone were crossed over from spironolactone to amiloride (146 patients). Amiloride use resulted in a remarkable reduction in BP levels of 20.4 mm Hg, comparable to the 18.3 mm Hg observed with spironolactone, suggesting that amiloride might be an effective alternative agent for these patients. Based on these findings, the European guidelines propose the use of amiloride as an alternative option if spironolactone is contraindicated or not well tolerated ^15^.

### Data from clinical studies of spironolactone versus central acting drugs

Although concrete evidence supports the superiority of mineralocorticoid receptor antagonists over alpha- and beta-blockers for patients with resistant hypertension, there is a lack of evidence regarding the use of central acting drugs in such patients. In this setting, the recent Resistant Hypertension Optimal Treatment (ReHOT) study compared the impact of spironolactone and clonidine in 187 patients with resistant hypertension ^16^. BP control assessed with office and 24-hour ambulatory BP monitoring was similar across the two groups of patients. However, the 24-hour systolic and diastolic BP reduction and the daytime diastolic BP reductions observed with spironolactone were significantly greater than those observed with clonidine. Given the easier dosage scheme of spironolactone and the greater benefits in various ambulatory BP parameters, spironolactone seems to be a preferable option over clonidine ^17^.

### Meta-analytic data of mineralocorticoid antagonists versus other drug classes

Important information emerged from meta-analytic data for the use of mineralocorticoid receptor antagonists in patients with resistant hypertension. In 869 patients from four trials, spironolactone as add-on therapy was associated with a reduction in BP of 16.67/6.11 mm Hg ^16^. A meta-analysis of 662 patients and five trials found that the addition of spironolactone in patients with resistant hypertension resulted in a reduction in office BP levels of 15.73/6.21 mm Hg compared with placebo, but compared with other drugs (beta-blocker, candesartan, or alpha methyldopa), spironolactone reduced home systolic BP by 4.5 mm Hg ^18^. In a meta-analysis of five studies and 553 patients with resistant hypertension, spironolactone reduced 24-hour, daytime, nighttime, and office BP by 10.50/4.09, 10.20/4.14, 10.02/3.21, and 16.99/6.18 mm Hg, respectively ^19^. Last, a meta-analysis of five studies and 755 patients with resistant hypertension found a greater reduction in systolic BP levels of 7.4 mm Hg (in the randomized studies) and 11.9 mm Hg (in the non-randomized studies) with mineralocorticoid receptor antagonists compared with other fourth-line options (bisoprolol, doxazosin, furosemide, or other renin–angiotensin system blockers) ^20^.

Collectively, accumulating evidence suggests that mineralocorticoid receptor antagonists are the optimal choice as the fourth-line option in patients with resistant hypertension, and data favor their use over central acting drugs, alpha- and beta-blockers. Most data come from studies with spironolactone. However, eplerenone may be considered as an alternative when adverse effects (such as gynecomastia or vaginal bleeding) are observed with spironolactone therapy ^21^, although strong data with eplerenone use are currently missing. Given the substantial fluid retention observed in these patients and the findings of the PATHWAY-2 study, amiloride is an alternative option, while treatment with doxazosin, bisoprolol, or clonidine may also be considered when either mineralocorticoid receptor antagonists or amiloride is contraindicated or adverse events occur.

## Interventional options for resistant hypertension

Interventional approaches represent a novel potential addition on lifestyle interventions and pharmacological therapy for the management of resistant hypertension. The neurogenic mechanisms implicated in BP elevation have been the target of several interventional approaches such as renal sympathetic denervation (RSD) and carotid baroreceptor activation therapy ^22– 24^. The resistance of the arterial tree walls is another important factor contributing to the rise of BP. Recently, the creation of arteriovenous anastomosis between the distal iliac vein and artery to add a low resistance compartment to the arterial tree was investigated in patients with resistant hypertension ^25^.

### Renal sympathetic denervation

The early RSD studies SYMPLICITY HTN-1 ^26^ and -2 ^27^ showed impressive reductions in BP and created high expectations for the future of the procedure in the hypertension treatment field. However, the first randomized sham-controlled study (SYMPLICITY HTN-3) failed to show any significant benefits of RSD over sham control in the reduction of both office and ambulatory BP in patients with drug-resistant hypertension ^28^, and the technique fell into hibernation. Although several pitfalls were identified, the incomplete denervation and lack of circumferential, four-quadrant sympathetic fiber interruption were the main factors pointed out as causes of the negative findings of the study ^29^. Three recent randomized sham-controlled studies used improved technologies and techniques to achieve optimal renal denervation and offered encouraging results.

The first one was the SPYRAL HTN-OFF MED trial, a randomized sham-controlled study of patients with untreated hypertension. Patients were randomly assigned to RSD of all accessible renal arterial vessels with the Symplicity Spyral multi-electrode catheter or the Symplicity G3 S (n = 38) or a sham procedure (n = 42). After 3 months, RSD resulted in significantly greater reductions in office and ambulatory BP levels compared with those of the sham control group (BP reduction differences of −7.7/−4.9 mm Hg and −5.0/−4.4 mm Hg in office and ambulatory BP, respectively). Importantly, no patient reported any safety concerns ^30^. Similar results were observed in the SPYRAL HTN-ON MED trial, a randomized sham control study (of patients with uncontrolled hypertension on one to three anti-hypertensive drugs) that used the same techniques described for SPYRAL-OFF MED. RSD resulted in a greater reduction in office and ambulatory BP compared with the sham technique group (BP reduction differences of −6.8/−3.5 mm Hg and −7.4/−4.1 mm Hg in office and ambulatory BP, respectively). No reports of renal artery stenosis or worsening of renal function were reported ^31^. The RADIANCE-HTN SOLO study was the third study that examined whether RSD performed with endovascular ultrasound reduces ambulatory BP in patients with hypertension in the absence of anti-hypertensive medication. After 2 months, significantly greater decreases in BP of −6.5/−4.1, −4.1/−1.8, and −7.1/−3.6 mm Hg in office, ambulatory, and home BP were reported with RSD compared with the sham control group ^32^.

A recent study assessed the efficacy of different ablation methods in reducing BP ^33^. Particularly, patients with resistant hypertension were assigned to either treatment with radiofrequency RSD of the main renal arteries, side branches, and accessories or an endovascular ultrasound-based RSD of the main renal artery. After 3 months, BP levels were significantly more reduced in the ultrasound ablation group compared with the radiofrequency ablation group of the main renal artery (−13.2 ± 13.7 versus −6.5 ± 10.3 mm Hg, respectively). Importantly, no significant difference was found between the radiofrequency ablation groups (−8.3 ± 11.7 mm Hg in the additional side branch ablation) or between the ultrasound and the side branch ablation groups, suggesting potential superiority of ultrasound over radiofrequency ablation of the main artery in reducing BP levels ^33^. However, larger studies are needed to confirm or dispute the superiority of one technique over the other. Up to then, no approach could be considered the preferred or first-choice option.

Collectively, these studies resurfaced the clinical and research interest for RSD in the management of resistant hypertension. Although more patients demonstrated BP reduction with RSD than with the sham procedure, in several study participants who underwent RSD, an increase in BP was noticed, suggesting that some patients are not eligible for RSD. The quest of patient eligibility requires the identification of reliable and accurate predictors of BP response, a very demanding step with unknown outcome. Studies with larger study populations and longer follow-up periods are needed to establish the safety and efficacy of the technique, and several such studies are either planned or being conducted. The results of these studies will either re-enforce the concept of RSD or put the final nail in the coffin of this interventional approach.

### Iliac vein and artery anastomosis

The ROX CONTROL HTN study randomly assigned 83 patients with resistant hypertension to either pharmacological treatment plus placement of an arteriovenous coupler or pharmacological therapy alone. After 6 months, significant reductions in office and ambulatory systolic BP of 26.9 and 13.5 mm Hg were noted with the anastomosis device group, respectively. In contrast, such benefits were not observed in the control group (3.7 and 0.5 mm Hg, respectively). Similarly, a significant reduction in diastolic BP was reported with the device. However, implantation of the arteriovenous coupler was associated with ipsilateral venous stenosis in 29% of the patients, who received venoplasty or stenting ^34^. After 12 months, office BP and ambulatory BP were reduced by 25/20.8 and 12.6/15.3 mm Hg, respectively, suggesting that the technique might offer long-lasting benefits in BP levels. Nevertheless, the percentage of patients who presented venous stenosis increased to 33%, who were treated successfully with venous stenting ^35^. In conclusion, although iliac anastomosis showed promise in terms of efficacy, the safety concerns were significant and this approach was recently abandoned.

### Carotid baroreceptor activation therapy

Carotid baroreceptor activation therapy is a device-based approach aiming to activate the baroreceptors that signal the brain to activate a sympatholytic response. Such approaches might be useful in conditions characterized by sympathetic overactivity, such as hypertension, heart failure, and arrhythmias ^36, 37^. The potential benefits might be due to the reduction of heart rate (and thus cardiac workload and energy demands) and the manifestation of arterial dilation, which results in reduction of the peripheral resistance and enhancement of renal blood flow and natriuresis.

The Rheos Pivotal Trial assessed the impact of the Rheos system, a device that uses electrical impulses from a pulse generator to chronically activate the baroreflex at the carotid sinus, on BP levels in 265 patients with resistant hypertension. In the first group of patients, treatment was applied for the first 6 months, whereas in the second group, a delayed treatment initiation was implemented at the 6-month visit; 42% of patients in the first group versus 24% in the second group achieved systolic BP of less than 140 mm Hg at 6 months, and more than 50% in both groups had a systolic BP of less than 140 mm Hg at 12 months. However, the procedural safety endpoints of the study were not met since procedural complications occurred in 25% of the patients (transient or permanent nerve injury or general surgical complications) ^38^.

However, the same company developed a second-generation device of smaller size, the Barostim Neo, which uses a smaller electrode on the surface of only one of the sinuses, thus reducing the invasiveness of the procedure and extending the battery life and replacement period ^39^. The Barostim Neo System in the Treatment of Heart Failure/Barostim Hope for Heart Failure and the recent Baroreflex Activation Therapy in Patients with Heart Failure and a Reduced Ejection Fraction trials ^40, 41^ demonstrated significant benefits in the functional status, quality of life, and exercise capacity in patients with heart failure and reduced injection fraction. Importantly, these studies met the safety endpoints; thus, the device was granted approval by the US Food and Drug Administration ^39^. The device has also been approved in Europe for the management of resistant hypertension; the Barostim Neo trial demonstrated a persistent reduction in BP after 6 months (of a systolic BP of approximately 26 mm Hg) and an adequate safety profile ^39, 42^.

Overall, baroreflex activation therapy is approved for the treatment of resistant hypertension in Europe and for the treatment of heart failure with reduced ejection fraction in the US. However, it is more invasive than RSD, the safety of the procedure is not unequivocally proven, and thus it has not gained either wide application or general acceptance by hypertensive experts for the management of resistant hypertension.

The MobiusHD carotid bulb expansion device is an under-examination device that is used to reduce BP through stretching of the carotid artery at the bulb, which in turn activates the carotid baroreceptors. The Controlling and Lowering Blood Pressure with the MOBIUSHD (CALM-FIM_EUR) study recently reported pronounced reduction in BP levels of 24/12 mm Hg in office and 21/12 mm Hg in ambulatory BP with the use of the MobiusHD device in patients with resistant hypertension ^43^. Importantly, the device demonstrated an acceptable safety profile ^43^. Two other trials—the CALM-FIM_US ^44^ and the Controlling and Lowering Blood Pressure with the MobiusHD (CALM-2) ^45^ studies—are examining the use of the MobiusHD device in patients with resistant hypertension. The results of these studies are eagerly awaited to further clarify the efficacy and safety of this approach and strengthen its role in the management of resistant hypertension.

## Continuous positive airway pressure therapy and resistant hypertension

Obstructive sleep apnea (OSA) is highly prevalent in patients with resistant hypertension ^46– 51^. It has been suggested that the increased fluid retention and consequent upper airway edema may explain the high prevalence of OSA in these patients ^52, 53^. In addition, central fluid accumulation during sleep seems to significantly contribute to the manifestation and worsening of OSA ^54– 56^. Treatment of OSA with continuous positive airway pressure (CPAP) in patients with resistant hypertension was found to induce a modest but significant reduction in BP levels. In a study of patients with resistant hypertension and OSA, CPAP treatment resulted in a reduction in ambulatory BP of 3.1/3.2 mm Hg, an effect that was even greater in patients more adherent to CPAP therapy (reduction in ambulatory BP of 4.4/4.1 mm Hg with at least 4 hours of CPAP treatment per night) ^57^. In contrast, a favorable impact of CPAP treatment on the prevention of cardiovascular events has not yet been demonstrated ^58^.

## Refractory hypertension

The term refractory hypertension has been recently re-introduced and was included in the 2017 American guidelines for the management of hypertension ^59^. Refractory hypertension is defined as failure of BP control with the use of five or more anti-hypertensive drugs of different drug classes, including a long-acting thiazide diuretic, such as chlorthalidone, and a mineralocorticoid receptor antagonist ^59^. This novel type of uncontrolled hypertension seems to be rare, affecting less than 5% of the patients referred to a specialized clinic for uncontrolled hypertension. Furthermore, refractory hypertension was found to be more frequent in African-American, younger, and female patients ^60– 62^. There is evidence suggesting that the cause of treatment failure in patients with refractory hypertension, in contrast to resistant hypertension, is the increased sympathetic tone, rather than fluid retention, as indicated by the increased heart rate levels and urine norepinephrine excretion in such patients ^60– 62^. Therefore, intensification of diuretic therapy may not be effective, and sympatholytic agents or device-based therapy may be preferable ^60– 62^. However, data in such patients are still missing and studies are needed to identify optimal management options.

## Conclusions

During the past few years, important data for the management of resistant hypertension have emerged. Most data support that mineralocorticoid antagonists (and especially spironolactone) present more favorable BP-lowering properties in patients with resistant hypertension compared with central acting drugs, alpha- and beta-blockers. In case of contraindications or adverse events, amiloride should be used as an alternative option followed by doxazosin, bisoprolol, or clonidine.

Several device-based approaches are being investigated, and recent RSD trials have rekindled interest in the interventional therapy of resistant hypertension. The few studies implementing carotid baroreceptor stimulation have shown favorable results, which need to be verified in controlled trials with a long follow-up period, while safety concerns need to be adequately addressed. Iliac anastomosis devices are no longer available in our therapeutic armamentarium. Overall, we are living in exciting times in the resistant hypertension field, and a lot of data, especially about the role of interventional approaches in the treatment of resistant hypertension, are eagerly expected in the near future.

## References

[1] Williams B Mancia G Spiering W: 2018 ESC/ESH Guidelines for the management of arterial hypertension. *Eur Heart J.* 2018;39(33):3021–104. 10.1093/eurheartj/ehy339 30165516

[2] Daugherty SL Powers JD Magid DJ: Incidence and prognosis of resistant hypertension in hypertensive patients. *Circulation.* 2012;125(13):1635–42. 10.1161/CIRCULATIONAHA.111.068064 22379110 PMC3343635

[3] Mancia G Fagard R Narkiewicz K: 2013 ESH/ESC guidelines for the management of arterial hypertension: the Task Force for the Management of Arterial Hypertension of the European Society of Hypertension (ESH) and of the European Society of Cardiology (ESC). *Eur Heart J.* 2013;34(28):2159–219. 10.1093/eurheartj/eht151 23771844

[4] Williams B MacDonald TM Morant S: Spironolactone versus placebo, bisoprolol, and doxazosin to determine the optimal treatment for drug-resistant hypertension (PATHWAY-2): a randomised, double-blind, crossover trial. *Lancet.* 2015;386(10008):2059–68. 10.1016/S0140-6736(15)00257-3 26414968 PMC4655321

[5] Carey RM Calhoun DA Bakris GL: Resistant Hypertension: Detection, Evaluation, and Management: A Scientific Statement From the American Heart Association. *Hypertension.* 2018;72(5):e53–e90. 10.1161/HYP.0000000000000084 30354828 PMC6530990

[6] Bhatt H Siddiqui M Judd E: Prevalence of pseudoresistant hypertension due to inaccurate blood pressure measurement. *J Am Soc Hypertens.* 2016;10(6):493–9. 10.1016/j.jash.2016.03.186 27129931 PMC4905807

[7] Egan BM Zhao Y Li J: Prevalence of optimal treatment regimens in patients with apparent treatment-resistant hypertension based on office blood pressure in a community-based practice network. *Hypertension.* 2013;62(4):691–7. 10.1161/HYPERTENSIONAHA.113.01448 23918752 PMC4066303

[8] de Jager RL van Maarseveen EM Bots ML: Medication adherence in patients with apparent resistant hypertension: findings from the SYMPATHY trial. *Br J Clin Pharmacol.* 2018;84(1):18–24. 10.1111/bcp.13402 28815689 PMC5736834

[9] Chobanian AV Bakris GL Black HR: Seventh report of the Joint National Committee on Prevention, Detection, Evaluation, and Treatment of High Blood Pressure. *Hypertension.* 2003;42(6):1206–52. 10.1161/01.HYP.0000107251.49515.c2 14656957

[10] Aguilera MT de la Sierra A Coca A: Effect of alcohol abstinence on blood pressure: assessment by 24-hour ambulatory blood pressure monitoring. *Hypertension.* 1999;33(2):653–7. 10.1161/01.hyp.33.2.653 10024322

[11] Nishizaka MK Pratt-Ubunama M Zaman MA: Validity of plasma aldosterone-to-renin activity ratio in African American and white subjects with resistant hypertension. *Am J Hypertens.* 2005;18(6):805–12. 10.1016/j.amjhyper.2005.01.002 15925740

[12] He FJ MacGregor GA: Effect of longer-term modest salt reduction on blood pressure. *Cochrane Database Syst Rev.* 2004; (3):CD004937. 10.1002/14651858.CD004937 15266549

[13] Boudville N Ward S Benaroia M: Increased sodium intake correlates with greater use of antihypertensive agents by subjects with chronic kidney disease. *Am J Hypertens.* 2005;18(10):1300–5. 10.1016/j.amjhyper.2004.08.031 16202852

[14] Manolis AJ Poulimenos LE Kallistratos MS: Sympathetic overactivity in hypertension and cardiovascular disease. *Curr Vasc Pharmacol.* 2014;12(1):4–15. 10.2174/15701611113119990140 23905597

[15] Williams B MacDonald TM Morant SV: Endocrine and haemodynamic changes in resistant hypertension, and blood pressure responses to spironolactone or amiloride: the PATHWAY-2 mechanisms substudies. *Lancet Diabetes Endocrinol.* 2018;6(6):464–75. 10.1016/S2213-8587(18)30071-8 29655877 PMC5966620

[16] Krieger EM Drager LF Giorgi DMA: Spironolactone Versus Clonidine as a Fourth-Drug Therapy for Resistant Hypertension: The ReHOT Randomized Study (Resistant Hypertension Optimal Treatment). *Hypertension.* 2018;71(4):681–90. 10.1161/HYPERTENSIONAHA.117.10662 29463627

[17] Zhao D Liu H Dong P: A meta-analysis of add-on use of spironolactone in patients with resistant hypertension. *Int J Cardiol.* 2017;233:113–7. 10.1016/j.ijcard.2016.12.158 28089457

[18] Liu L Xu B Ju Y: Addition of spironolactone in patients with resistant hypertension: A meta-analysis of randomized controlled trials. *Clin Exp Hypertens.* 2017;39(3):257–63. 10.1080/10641963.2016.1246564 28448185

[19] Wang C Xiong B Huang J: Efficacy and Safety of Spironolactone in Patients with Resistant Hypertension: A Meta-analysis of Randomised Controlled Trials. *Heart Lung Circ.* 2016;25(10):1021–30. 10.1016/j.hlc.2016.02.016 27118266

[20] Sinnott SJ Tomlinson LA Root AA: Comparative effectiveness of fourth-line anti-hypertensive agents in resistant hypertension: A systematic review and meta-analysis. *Eur J Prev Cardiol.* 2017;24(3):228–38. 10.1177/2047487316675194 27856806

[21] Calhoun DA: Advances in resistant hypertension. *Ann Transl Med.* 2018;6(15):294. 10.21037/atm.2018.07.21 30211182 PMC6123198

[22] Schlaich MP: Renal Sympathetic Denervation: A Viable Option for Treating Resistant Hypertension. *Am J Hypertens.* 2017;30(9):847–56. 10.1093/ajh/hpx033 28338871

[23] Bolignano D Coppolino G: Baroreflex stimulation for treating resistant hypertension: ready for the prime-time? *Rev Cardiovasc Med.* 2018;19(3):89–95. 10.31083/j.rcm.2018.03.3185 31054557

[24] Voora R Hinderliter AL: Modulation of Sympathetic Overactivity to Treat Resistant Hypertension. *Curr Hypertens Rep.* 2018;20(11):92. 10.1007/s11906-018-0893-8 30194545

[25] Schlaich MP Azzam O Sata Y: Hypertension on the ROX: Durable Blood Pressure Lowering With Central Iliac Arteriovenous Anastomosis. *Hypertension.* 2017;70(6):1084–6. 10.1161/HYPERTENSIONAHA.117.10246 29061721

[26] Krum H Schlaich M Whitbourn R: Catheter-based renal sympathetic denervation for resistant hypertension: a multicentre safety and proof-of-principle cohort study. *Lancet.* 2009;373(9671):1275–81. 10.1016/S0140-6736(09)60566-3 19332353

[27] Symplicity HTN-2 Investigators, Esler MD Krum H: Renal sympathetic denervation in patients with treatment-resistant hypertension (The Symplicity HTN-2 Trial): a randomised controlled trial. *Lancet.* 2010;376(9756):1903–9. 10.1016/S0140-6736(10)62039-9 21093036

[28] Bhatt DL Kandzari DE O'Neill WW: A controlled trial of renal denervation for resistant hypertension. *N Engl J Med.* 2014;370(15):1393–401. 10.1056/NEJMoa1402670 24678939

[29] Papademetriou V Stavropoulos K Doumas M: Now That Renal Denervation Works, How Do We Proceed? *Circ Res.* 2019;124(5):693–5. 10.1161/CIRCRESAHA.119.314695 30817252

[30] Townsend RR Mahfoud F Kandzari DE: Catheter-based renal denervation in patients with uncontrolled hypertension in the absence of antihypertensive medications (SPYRAL HTN-OFF MED): a randomised, sham-controlled, proof-of-concept trial. *Lancet.* 2017;390(10108):2160–70. 10.1016/S0140-6736(17)32281-X 28859944

[31] Kandzari DE Böhm M Mahfoud F: Effect of renal denervation on blood pressure in the presence of antihypertensive drugs: 6-month efficacy and safety results from the SPYRAL HTN-ON MED proof-of-concept randomised trial. *Lancet.* 2018;391(10137):2346–55. 10.1016/S0140-6736(18)30951-6 29803589

[32] Azizi M Schmieder RE Mahfoud F: Endovascular ultrasound renal denervation to treat hypertension (RADIANCE-HTN SOLO): a multicentre, international, single-blind, randomised, sham-controlled trial. *Lancet.* 2018;391(10137):2335–45. 10.1016/S0140-6736(18)31082-1 29803590

[33] Fengler K Rommel KP Blazek S: A Three-Arm Randomized Trial of Different Renal Denervation Devices and Techniques in Patients With Resistant Hypertension (RADIOSOUND-HTN). *Circulation.* 2019;139(5):590–600. 10.1161/CIRCULATIONAHA.118.037654 30586691

[34] Lobo MD Sobotka PA Stanton A: Central arteriovenous anastomosis for the treatment of patients with uncontrolled hypertension (the ROX CONTROL HTN study): a randomised controlled trial. *Lancet.* 2015;385(9978):1634–41. 10.1016/S0140-6736(14)62053-5 25620016

[35] Lobo MD Ott C Sobotka PA: Central Iliac Arteriovenous Anastomosis for Uncontrolled Hypertension: One-Year Results From the ROX CONTROL HTN Trial. *Hypertension.* 2017;70(6):1099–105. 10.1161/HYPERTENSIONAHA.117.10142 29061728

[36] Devgun J Jobanputra YB Arustamyan M: Devices and interventions for the prevention of adverse outcomes of tachycardia on heart failure. *Heart Fail Rev.* 2018;23(4):507–16. 10.1007/s10741-018-9680-5 29430580

[37] van Kleef MEAM Bates MC Spiering W: Endovascular Baroreflex Amplification for Resistant Hypertension. *Curr Hypertens Rep.* 2018;20(5):46. 10.1007/s11906-018-0840-8 29744599 PMC5942348

[38] Bisognano JD Bakris G Nadim MK: Baroreflex activation therapy lowers blood pressure in patients with resistant hypertension: results from the double-blind, randomized, placebo-controlled rheos pivotal trial. *J Am Coll Cardiol.* 2011;58(7):765–73. 10.1016/j.jacc.2011.06.008 21816315

[39] Lohmeier TE Hall JE: Device-Based Neuromodulation for Resistant Hypertension Therapy. *Circ Res.* 2019;124(7):1071–93. 10.1161/CIRCRESAHA.118.313221 30920919 PMC6442942

[40] Abraham WT Zile MR Weaver FA: Baroreflex Activation Therapy for the Treatment of Heart Failure With a Reduced Ejection Fraction. *JACC Heart Fail.* 2015;3(6):487–96. 10.1016/j.jchf.2015.02.006 25982108

[41] Zile M Lindenfeld J Weaver FA: Baroreflex Activation Therapy (BAT) in Patients with Heart Failure and a Reduced Ejection Fraction (HFrEF): The BeAT-HF Trial.2019 Reference Source

[42] Hoppe UC Brandt MC Wachter R: Minimally invasive system for baroreflex activation therapy chronically lowers blood pressure with pacemaker-like safety profile: results from the Barostim *neo* trial. *J Am Soc Hypertens.* 2012;6(4):270–6. 10.1016/j.jash.2012.04.004 22694986

[43] Spiering W Williams B Van der Heyden J: Endovascular baroreflex amplification for resistant hypertension: a safety and proof-of-principle clinical study. *Lancet.* 2017;390(10113):2655–61. 10.1016/S0140-6736(17)32337-1 28870716

[44] ClinicalTrials.gov: Controlling and Lowering Blood Pressure With The MOBIUSHD™ (CALM-FIM_US). Accessed February 9, 2020. Reference Source

[45] ClinicalTrials.gov: CALM- 2 - Controlling and Lowering Blood Pressure With the MobiusHD™ (CALM-2). Accessed February 9, 2020. Reference Source

[46] Lloberes P Lozano L Sampol G: Obstructive sleep apnoea and 24-h blood pressure in patients with resistant hypertension. *J Sleep Res.* 2010;19(4):597–602. 10.1111/j.1365-2869.2010.00839.x 20545837

[47] Logan AG Perlikowski SM Mente A: High prevalence of unrecognized sleep apnoea in drug-resistant hypertension. *J Hypertens.* 2001;19(12):2271–7. 10.1097/00004872-200112000-00022 11725173

[48] Min HJ Cho YJ Kim CH: Clinical Features of Obstructive Sleep Apnea That Determine Its High Prevalence in Resistant Hypertension. *Yonsei Med J.* 2015;56(5):1258–65. 10.3349/ymj.2015.56.5.1258 26256968 PMC4541655

[49] Muxfeldt ES Margallo VS Guimarães GM: Prevalence and associated factors of obstructive sleep apnea in patients with resistant hypertension. *Am J Hypertens.* 2014;27(8):1069–78. 10.1093/ajh/hpu023 24705438

[50] Walia HK Li H Rueschman M: Association of severe obstructive sleep apnea and elevated blood pressure despite antihypertensive medication use. *J Clin Sleep Med.* 2014;10(8):835–43. 10.5664/jcsm.3946 25126027 PMC4106935

[51] Pratt-Ubunama MN Nishizaka MK Boedefeld RL: Plasma aldosterone is related to severity of obstructive sleep apnea in subjects with resistant hypertension. *Chest.* 2007;131(2):453–9. 10.1378/chest.06-1442 17296647

[52] Di Murro A Petramala L Cotesta D: Renin-angiotensin-aldosterone system in patients with sleep apnoea: prevalence of primary aldosteronism. *J Renin Angiotensin Aldosterone Syst.* 2010;11(3):165–72. 10.1177/1470320310366581 20488824

[53] Prejbisz A Florczak E Klisiewicz A: Relationship between primary aldosteronism and obstructive sleep apnoea, metabolic abnormalities and cardiac structure in patients with resistant hypertension. *Endokrynol Pol.* 2013;64(5):363–7. 10.5603/EP.2013.0019 24186593

[54] Friedman O Bradley TD Chan CT: Relationship between overnight rostral fluid shift and obstructive sleep apnea in drug-resistant hypertension. *Hypertension.* 2010;56(6):1077–82. 10.1161/HYPERTENSIONAHA.110.154427 21060007

[55] Chiu KL Ryan CM Shiota S: Fluid shift by lower body positive pressure increases pharyngeal resistance in healthy subjects. *Am J Respir Crit Care Med.* 2006;174(12):1378–83. 10.1164/rccm.200607-927OC 16998093

[56] Redolfi S Arnulf I Pottier M: Effects of venous compression of the legs on overnight rostral fluid shift and obstructive sleep apnea. *Respir Physiol Neurobiol.* 2011;175(3):390–3. 10.1016/j.resp.2011.01.001 21220055

[57] Martínez-García MA Capote F Campos-Rodríguez F: Effect of CPAP on blood pressure in patients with obstructive sleep apnea and resistant hypertension: the HIPARCO randomized clinical trial. *JAMA.* 2013;310(22):2407–15. 10.1001/jama.2013.281250 24327037

[58] McEvoy RD Antic NA Heeley E: CPAP for Prevention of Cardiovascular Events in Obstructive Sleep Apnea. *N Engl J Med.* 2016;375(10):919–31. 10.1056/NEJMoa1606599 27571048

[59] Whelton PK Carey RM Aronow WS: 2017 ACC/AHA/AAPA/ABC/ACPM/AGS/APhA/ASH/ASPC/NMA/PCNA Guideline for the Prevention, Detection, Evaluation, and Management of High Blood Pressure in Adults: A Report of the American College of Cardiology/American Heart Association Task Force on Clinical Practice Guidelines. *Hypertension.* 2018;71(19):e127–e248. 10.1016/j.jacc.2017.11.006 29146535

[60] Dudenbostel T Acelajado MC Pisoni R: Refractory Hypertension: Evidence of Heightened Sympathetic Activity as a Cause of Antihypertensive Treatment Failure. *Hypertension.* 2015;66(1):126–33. 10.1161/HYPERTENSIONAHA.115.05449 25987662 PMC4465856

[61] Siddiqui M Calhoun DA: Refractory versus resistant hypertension. *Curr Opin Nephrol Hypertens.* 2017;26(1):14–9. 10.1097/MNH.0000000000000286 27798457

[62] Velasco A Siddiqui M Kreps E: Refractory Hypertension Is not Attributable to Intravascular Fluid Retention as Determined by Intracardiac Volumes. *Hypertension.* 2018;72(2):343–9. 10.1161/HYPERTENSIONAHA.118.10965 29866740 PMC6043380

